# Altered glycobiology of stem cells linked to age-related osteoarthritis

**DOI:** 10.18632/aging.100356

**Published:** 2011-07-29

**Authors:** Subramanya Srikantan, Myriam Gorospe

**Affiliations:** ^1^ Laboratory of Molecular Biology and Immunology, National Institute on Aging-Intramural Research Program, National Institutes of Health, Baltimore, MD 21224

A decline in cellular homeostasis in older individuals underlies age-related pathologies like osteoporosis and osteoarthritis. In this issue of *Impact Aging*, Jiang et al. [1] report key differences in the patterns of expressed mRNAs in bone-marrow mesenchymal stem cells (bmMSCs) of young donors compared with old human donors. The distinct subsets of expressed genes associated with glycobiology are consistent with the underlying age-related decline in bone marrow function.

It is now well established that in older individuals stem cells can become “aged” and thus incapable of renewing surrounding tissues and organs as efficiently as young individuals. Experimental and clinical evidence has revealed the importance of stem cell aging in bone marrow transplants, as recipients of bone marrow from older donors do not fare as well as recipients of bone marrow from younger donors [2, 3]. However, the molecular mechanisms governing stem cell aging are not well understood. An important first step towards this goal is to delineate the gene expression differences between stem cells from young and old individuals. Bone marrow stem cells are particularly well suited for such studies, as they are relatively easy to purify to homogeneity. Jiang et al (2011) investigated mRNA expression profiles of bmMSCs from normal individuals and osteoarthritis patients and analyzed the data to address age- and osteoarthritis-related changes in mRNAs expressed in these cells. Apart from the expected differences in the expression of proliferation and cell cycle-related genes, bmMSCs showed age-increases in the expression of genes associated with the degradation of N-glycans and glycosaminoglycans and with the biosynthesis of glycosphingolipids. These results reveal major differences in the glycobiology and glycan compostion of young and old bmMSCs, associated with age-related changes in the cellular responses to autocrine and paracrine signals. The difference in glycan pathways may not be limited to bmMSCs or even to stem cells, but could be more widely prevalent among other cell types. For example changes in brain glycoprotein composition were reported in aging brain [4].

Gene expression analyses similar to that undertaken by Jiang and coworkers can provide critical insight into the molecular mechanisms of aging. The authors examine the implication of their findings by testing the ability of bmMSCs to respond to Na_2_SO_4_, a treatment that increases the sulfation of proteoglycans. BmMSCs responded to Na_2_SO_4_ treatment by inducing DNA synthesis, but the response was stronger in bmMSCs from aged donors, correlating with higher *SULF1* mRNA expression levels. Further studies are required to address how Na_2_SO_4_ treatment affects DNA synthesis, whether *SULF1* is implicated in this differential response, and why the response is different in adult and aged bmMSCs. The report also sets the stage for more studies of the molecular mechanism whereby changes in the expression of other glycan pathway genes affect aging. For example *HEXA* and *HEXB* mRNAs, encoding two hexoseaminidases important for the degradation of glycosphingolipids, were upregulated in aged bmMSCs. Further investigation will reveal whether this differential increase impacts upon lipid raft formation in older cells and thus affects cellular signaling. In this regard, it was proposed earlier that hexoseaminidase inhibitors could be exploited for the treatment of osteoarthritis [5].

In sum, studying differences in gene expression in persons of different ages can reveal specific molecular signatures of physiologic and pathologic states. Accordingly, the patterns of expressed genes are increasingly recognized as providing valuable frameworks for the diagnosis and management of age-related diseases.
